# 

**Published:** 1862-06

**Authors:** 


### CONTENTS.

ORIGINAL ESSAYS AND COMMUNICATIONS.

Dental Fees. By J. Taft .......... 245
Success in the Dental Profession. No. 2. By C. C. Dills, D. D. S .......... 249

PROCEEDINGS OF SOCIETIES.

Extracts from the Proceedings of the Odontological Society, London .......... 251

SELECTIONS.

Necrosis of Inferior Maxilla, involving the Whole Bone .......... 260
Periodontitis .......... 261
Anæsthetics in Military Surgery .......... 265
Experiments in Vulcanizing Rubber Compounds .......... 268
Bibliographical .......... 271
Toxicology .......... 278
Trochisci Calami .......... 281
Removal of a Tumor involving the Parotid and Submaxillary Glands .......... 282
The Influence of Sleep over Disease .......... 285

EDITORIAL.

Concentration .......... 287
Can't Afford it .......... 288
Persulphate of Iron .......... 289
Foil .......... ib.
Book Notice .......... 290

NOTICES.

Indiana State Dental Association .......... 290
The American Dental Convention, .......... 291
The American Dental Association, .......... 292

